# Characterization of Levan Fructan Produced by a *Gluconobacter japonicus* Strain Isolated from a Sugarcane Processing Facility

**DOI:** 10.3390/microorganisms12010107

**Published:** 2024-01-05

**Authors:** Gillian O. Bruni, Yunci Qi, Evan Terrell, Rebecca A. Dupre, Christopher P. Mattison

**Affiliations:** 1 U.S. Department of Agriculture, Agricultural Research Service, Southern Regional Research Center, New Orleans, LA 70124, USA; 2 U.S. Department of Energy, Oak Ridge Institute for Science and Education, Oak Ridge, TN 37831, USA

**Keywords:** levan fructan, exopolysaccharide, levansucrase, sugarcane, sugar processing

## Abstract

During raw sugarcane processing, a significant portion of lost sucrose is attributable to microbial degradation. Sucrose consumption by many bacteria is also linked to the production of exopolysaccharides (EPS) such as dextrans and fructans. These resulting EPS cause operational challenges during raw sugar manufacturing. Here, we report the characterization of EPS from a fructan-forming *Gluconobacter japonicus* bacterium that we previously isolated from a Louisiana sugarcane factory. The genome sequencing revealed the presence of two encoded levansucrase genes, *lsrA* and *lsrB*. One levansucrase, LsrB, was detected in the secreted protein fraction of *G. japonicus* LASM12 by QTOF LC-MS. The spotting assays indicated that *G. japonicus* produces EPS using sucrose and raffinose as substrates. The *G. japonicus* EPS correlated with levan fructan commercial standards by ^1^H-NMR, and with the characteristic carbohydrate fingerprint region for FTIR spectra, confirming that the *G. japonicus* EPS is levan fructan. The glycosyl composition and glycosyl linkage analysis revealed a linear β-2,6-fructofuranosyl polysaccharide with occasional (5.7%) β-2,1-fructofuranosyl branches. The gel permeation chromatography of the levan fructan EPS showed two main peaks at 4.5 kDa and 8 kDa and a very minor peak at 500 kDa. *G. japonicus* was identified as a producer of levan fructan. These findings will be useful for future studies aimed at evaluating the impact of levan fructans on sugar crop processing, which have been historically underestimated in industry.

## 1. Introduction

During post-harvest sugarcane processing, a significant fraction of lost sucrose is attributable to microbial degradation by both bacteria and yeast that readily consume sucrose [1,2,3]. Sucrose consumption by many bacteria is also linked to the production of exopolysaccharides (EPS) such as dextrans and levan fructans by the activity of dextransucrases and levansucrases, respectively. Fructans are homopolymers composed of fructose monosaccharides and are produced by various plants and microbes. Fructans may function as a storage carbohydrate and function in abiotic stress resistance [4]. Fructan polysaccharides comprising β-2,1-linked fructofuranose residues are referred to as inulins and are produced by inulosucrases. Levan fructan is composed of β-2,6-linked fructofuranose residues in a linear chain with intermittent β-2,1-linked side chains [5] and are produced by a levansucrase that catalyzes the hydrolysis of the sucrose disaccharide and transfer of the fructosyl residue to a growing fructan polymer [6,7]. Levans are also produced by various grasses such as *Phleum pratense* (Timothy grass), *Dactylis glomerata* (Cat grass), *Agropyron cristatum* (Crested wheatgrass) as well as several microbes [8].

We previously identified *Gluconobacter* isolates from Louisiana sugarcane mills and reported that these strains readily consume sucrose and produce copious amounts of EPS when grown on sucrose as a carbon source [3,9]. As *Gluconobacter* strains have previously been reported to produce levan [6,10], these isolates were of interest as potential levan-producing bacteria present in sugarcane mills. Recent microbial profiling of sugarcane mill samples by amplicon sequencing has revealed the average relative abundance of the family *Acetobacteraceae* (to which *Gluconobacter* belongs) was 3.9% [11]. These results also revealed the presence of *Zymomonas* bacteria in juices which have also been reported to encode levansucrases [11,12,13,14]. While it appears that *Gluconobacter* bacteria may contribute to the overall production of levan fructan EPS during sugarcane processing, given the low frequency of this microbe in sugarcane juice fractions [11], it is likely that other bacteria also contribute to previously underestimated fructan levels during sugar crop processing as well [15]. Additional microorganisms capable of producing fructans include *Weissella,* which has been reported to produce multiple types of EPS including dextrans, levan, and inulin EPS depending on the isolate [16]. In addition, various other bacteria have been reported as sources of levansucrase enzymes, as listed on the carbohydrate active enzymes (CAZy) database [17,18,19,20,21]. Notably, several of these such as *Pantoea*, *Leuconostoc*, and *Lactobacillus* were also detected during a microbiome analysis of sugarcane factory juices [11].

In contrast to dextran, the extent of the impact that levan-producing organisms have during the processing of sugarcane is poorly understood [15,22]. In recent years, there has been increased interest surrounding the presence of fructans found in factory juices during raw sugarcane processing. It has been suggested that these fructans have been historically underestimated [15,22]. Furthermore, the widely-used haze method used to estimate the dextrans in juices does not distinguish between dextrans and fructans, but is based on an insoluble “haze”. Therefore, it is unclear to what extent the existing methods may have underestimated the presence of bacterial-derived fructan exopolysaccharides. Despite the interest in the presence and potential impact of fructans on sugarcane processing, little has been reported on the identity of the fructan-producing microbes until recently [11]. This study provides one of the earliest isolations and bona fide confirmations of fructan production by a microbial isolate from a Louisiana sugarcane processing facility.

Generally, operational problems caused by sucrose inversion and EPS production during sugar manufacturing include increased viscosity, the clogging of filters, elongated sugar crystals, and overall decreased sugar recovery, resulting in significant economic loss to the sugar industry [23]. Additionally, fructans have many potential applications in cosmetics, medicine, industry, and biotechnology, including wound healing and immunostimulating activity [24,25,26]. For example, in addition to having a potential impact during sugar crop processing, fructan polysaccharides have been purposed for numerous biotechnological and medical uses, including immunomodulation [27], the pro-apoptotic treatment of neuroblastoma cells [5,28], and as an influence on digestion and gut microbiota as a prebiotic [5,25,29]. Therefore, there is ongoing interest in identifying various microbial levansucrases and the resulting polysaccharides for the development of various bio-based products and applications, since different levansucrases produce levans with a diverse range of sizes and branching [8,30].

In this study, we sought to further characterize levan fructan EPS produced by a *Gluconobacter* isolate that is relevant to sugarcane processing. Here, we report the identification of two levansucrase enzymes encoded in the genome of the *G. japonicus* strain LASM12, which we designated as LsrA and LsrB. LsrB was detected in the secreted protein fraction and is most likely involved in the synthesis of levan fructan polysaccharide. We also report the average polymer size, glycosyl composition, and glycosidic linkages of the *Gluconobacter*-derived levan fructan EPS.

## 2. Materials and Methods

### 2.1. G. japonicus LASM12 Levansucrase Sequence Analysis

The draft genome of *G. japonicus* LASM12 was queried for homologs of the levansucrase enzyme encoded by *Gluconobacter albidus* strain TMW2.1191 [6] using tBLASTn. The amino acid sequences of the two putative levansucrases were aligned with ClustalOmega version 1.2.2 against previously characterized GH68 enzymes from other Gram-negative bacteria listed on the CAZy database as of November 2022. One levansucrase from the Gram-positive *Leuconostoc mesenteroides* (LevS; AAY19523) was used as an outgroup. A maximum likelihood tree was generated using PhyML version 3.3.20180621 [31]. Gaps present in >50% of sequences were masked from the alignment and the JTT substitution matrix was used [32]. Branches supported by less than 25% of 500 bootstraps were collapsed. These analyses were performed in Geneious version 2022.0.2 (Dotmatics, Boston, MA, USA).

### 2.2. Culturing and EPS Isolation

*G. japonicus* strain LASM12 was isolated and identified as previously described [9]. For EPS preparation, LASM12 was grown aerobically in TSY medium containing 10% sucrose in Erlenmeyer flasks and incubated at 28 °C for 5 days with 225 rpm shaking. Cells were removed by centrifugation at 10,000× *g* and EPS was precipitated from the supernatant at 20,000× *g* with three volumes of ice-cold absolute ethanol. Precipitated EPS was then lyophilized and stored under desiccation prior to analysis.

### 2.3. Bacterial Culturing for Precipitation of Secreted Proteins

*G. japonicus* LASM12 was inoculated into 50 mL TSY medium containing 50 g/L sucrose and grown for 72 h at 28 °C, at 225 rpm [33]. Bacterial cells were removed by centrifugation at 8,800× *g*, for 15 min at 4 °C. Proteins were precipitated from the supernatants by gradually adding ammonium sulfate up to 50% and stirring for 4 h, followed by overnight incubation at 4 °C, and precipitated proteins were pelleted at 24,000× *g*, for 20 min at 4 °C. Protein pellets were solubilized in 50 mM Tris HCl buffer containing protease inhibitor cocktail (Sigma-Aldrich, St. Louis, MO, USA). Protein samples were dialyzed in Slide-A-Lyzer dialysis cassettes 2,000 Da MWCO (Thermoscientific, Waltham, MA, USA) against 50 mM Tris HCl to remove excess salt and concentrated in Amicon Ultra-4 spin columns 10,000 Da MWCO (Millipore, Burlington, MA, USA).

### 2.4. Methods for MS-Based Identification of Secreted EPS-Forming Enzyme

Protein samples were run under reducing conditions on Novex Wedgewell 8% tris-glycine gels (Invitrogen, Grand Island, NY, USA) and stained with SimplyBlue Safe Stain (Invitrogen, Grand Island, NY, USA). Pre-stained Precision Plus Dual protein standard was purchased from Bio-Rad (Hercules, CA, USA). Gels were imaged on a LI-COR Biosciences (Lincoln, NE, USA) Odyssey CLx infrared imaging system imager with Image Studio software version 1.0.20.

Protein bands were excised and prepared for MS analysis according to the Center for Mass Spectrometry and Proteomics, University of Minnesota (adapted from the EMBL Method) [34]. Briefly, Coomassie-stained protein bands were excised, de-stained, reduced, alkylated, in-gel trypsin digested, and extracted as previously described [34]. Pooled extracted peptides were dried in a speed vac (Eppendorf, Hamburg, Germany) and suspended in 20 µL of 5% formic acid. Digested samples were run on an Agilent 6520 Q-TOF LC/MS equipped with a Chip Cube interface and 1200 LC system. A water/acetonitrile (Fisher, LC/MS grade) gradient containing 0.1% formic acid and a Zorbax 300SB-C18 Large Capacity Chip (II) was used for chromatographic separation (Agilent, Santa Clara, CA, USA). The protein digests were analyzed twice; MS/MS precursor exclusion lists were generated for the second analyses to improve protein identification.

Data were processed and searched using Mascot Distiller software version 2.8.2 (Matrix Science, Boston, MA, USA). A protein database was constructed from the *G. japonicus* LASM12 genome [9]. Searches were performed with peptide and fragment mass tolerances set at 20 and 50 ppm, respectively. Carbamidomethylation of cysteine was set as a fixed modification, and oxidation of methionine was included as a variable modification. The digestion enzyme was specified as trypsin and up to two missed cleavages were allowed.

### 2.5. Glycosyl Composition Analysis

Glycosyl composition analysis was performed at the Complex Carbohydrate Research Center (CCRC at University of Georgia) by combined gas chromatography-mass spectrometry (GC-MS) of the per-O-trimethylsilyl (TMS) derivatives of the monosaccharide methyl glycosides produced from the sample by acidic methanolysis as described previously [35]. Briefly, the dry sample (200 µg) was heated with methanolic HCl in a sealed screw-top glass test tube for 16 h at 80 °C. After cooling and removal of the solvent under a stream of nitrogen, the samples were re-N-acetylated and dried again. The sample was then derivatized with Tri-Sil^®^ (Thermoscientific, Waltham, MA, USA) at 80 °C for 30 min. GC-MS analysis of the TMS methyl glycosides was performed on an Agilent 7890A GC interfaced to a 5975C MSD, using an Supelco Equity-1 fused silica capillary column (30 m × 0.25 mm ID). The GC temperature program conditions were as follows: initial temperature of 80 °C with 2 min hold; ramp at 20 °C/min to 140 °C with 2 min hold; ramp at 2 °C/min to 200 °C; ramp at 30 °C/min to 250 °C with five minute hold.

### 2.6. Glycosyl Linkage Analysis

Glycosyl linkage analysis was performed at the CCRC (UGA, Athens, GA, USA) by GC-MS of the partially methylated alditol acetates (PMAAs) derivatives produced from the samples. The procedure is a slight modification of the one described by Heiss et al. [36].

A total of 300 µL of anhydrous dimethyl sulfoxide (DMSO) was added to the sample (590 μg) and the contents were stirred overnight. Permethylation was achieved by two rounds of treatment with sodium hydroxide (NaOH) suspension (see below) (15 min) and iodomethane (25 min). The sodium hydroxide base solution was prepared as previously described [37]. Briefly, to NaOH (50% *w*/*w*, 100 µL) was added methanol (200 µL, MeOH), and the mixture was vortexed. Then, DMSO (2 mL) was added, and the base solution was vortexed and centrifuged. The supernatant solution was removed, and fresh DMSO was added. This was repeated 5 times. After the final extraction, DMSO (2 mL) was added to the NaOH pellet and the solution was vortexed. This final base solution (300 µL) was added to the sample, and the mixture was magnetically stirred for 15 min. Then, iodomethane (70 µL) was added, and the sample was stirred at room temperature for 20 min. A second round of base and then iodomethane was performed to ensure complete methylation.

The permethylated materials were then hydrolyzed with 2 M trifluoroacetic acid (TFA) for 2 h at 121 °C and dried down with isopropanol under a stream of nitrogen. The samples were then reduced with NaBD_4_ in 100 mM NH_4_OH (10 mg/mL) overnight, neutralized with glacial acetic acid, and dried with methanol. Finally, the samples were O-acetylated using acetic anhydride (250 µL) and concentrated TFA (250 µL) at 50 °C for 20 min. The samples were dried under nitrogen, reconstituted in dichloromethane, and washed with nanopure water before injection into GC-MS.

The resulting partially methylated alditol acetates (PMAAs) were analyzed on an Agilent 7890A GC interfaced to a 5975C MSD; separation was performed on a Supelco 2331 fused silica capillary column (30 m × 0.25 mm ID) with a temperature gradient as follows: initial temperature of 60 °C with one minute hold; ramp at 27.5 °C/min to 170 °C; ramp at 4 °C/min to 235 °C with two minute hold; ramp at 3 °C/min to 240 °C with twelve minute hold. The method is a derivation of the linkage method detailed by Heiss et al. [36].

### 2.7. Determination of Molecular Weight

Molecular weight measurement was performed at CCRC (UGA, Athens, GA, USA) and determined by size exclusion chromatography (SEC) with RI detection. The sample (1.0 mg) was dissolved in 1 mL 50 mM ammonium acetate solution by subjecting the resulting mixture to sequential heating and cooling steps as previously described [38]. Sample mixtures were slowly heated to about 80 °C and then allowed to slowly cool.

The method used for SEC was adapted from Yanagisawa et al. [39]. The SEC system used was Agilent 1260 Infinity, which consists of a quaternary pump (G7111B) with a built-in degasser, high-performance autosampler (G7129A), and a refractive index detector (G7162A). A Superose 6 column from Cytiva was used for the separation. The eluent was 50 mM ammonium acetate. SEC conditions were as follows: sample concentration of 1 mg/mL, injection volume of 100 µL and flow rate of 0.5 mL/min. The RI detector cells were kept at 35 °C.

### 2.8. H-NMR

A mass of 5 mg of EPS sample was dissolved in 1 mL D_2_O (99.9% D) and lyophilized. A total of 0.5 mL of D_2_O was then added to the sample and transferred to a 5 mm NMR tube. All NMR data were acquired at 25 °C on a Bruker Advance III spectrometer (^1^H, 600.06 MHz) equipped with a cryoprobe using standard pulse sequences. A ^1^H spectrum was acquired with spectral width of 9615.3 Hz, 32,768 data points and eight transients with total recycle delay of 1.5 s. Chemical shifts were referenced to DSS (δH = 0.00 ppm). The spectra were processed and analyzed with MestreNova (version 14.0.1-23559). NMR data visualization was performed using nmrglue for Python 3.8.5 with Jupyter Notebook version 6.1.4 [40].

### 2.9. Fourier Transform Infrared (FTIR) Spectroscopy

Samples were analyzed using a Thermo Scientific Attenuated Total Reflectance (ATR) FTIR Nicolet iS20 instrument at 4 cm^−1^ resolution with 32 scans from 4000 to 400 cm^−1^. In addition to the LASM12 isolate EPS samples, two levan standards were also analyzed. The first levan standard prepared from Timothy grass (*Phleum pratense*) was purchased from Neogen (Lansing, MI, USA), and the second levan standard prepared from *Erwinia herbicola* was purchased from Sigma-Aldrich (St. Louis, MO, USA). To account for potential intrasample heterogeneity, each EPS and standard was analyzed in duplicate with independently collected aliquots (although only one representative spectrum is shown per sample). Data processing and visualization were performed using Microsoft Excel version 2311 and Python 3.8.5 with Jupyter Notebook version 6.1.4.

## 3. Results

### 3.1. Species-Level Identification of Gluconobacter Isolates from a Sugarcane Processing Facility

The *Gluconobacter* isolates LASM11 and LASM12 were previously identified as a *Gluconobacter* spp. by sequencing the V4 region of their 16S rRNA genes [3]. After obtaining draft genome sequences [9], both strains were identified as *Gluconobacter japonicus* based on the average nucleotide identity (ANI) to the genomes of type strains in GenBank [41,42]. During this analysis, it was noticed that the strains LASM11 and LASM12 had identical DNA sequences across large contigs (>100 kb), strongly indicating that these strains were re-isolates of the same organism.

### 3.2. Analysis of G. japonicus EPS Production on Various Sugars

**Figure 1:**
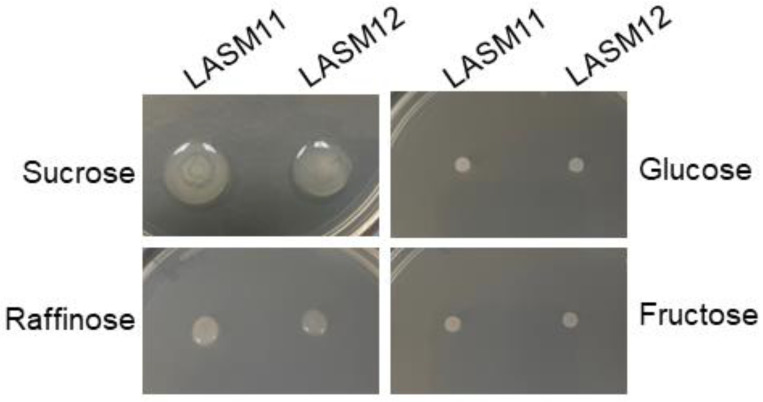
*G. japonicus* strains LASM11 and LASM12 utilize sucrose and raffinose as substrates for EPS formation. Overnight precultures were grown in MRS, normalized to 0.4 O.D., and 2.5 µL were spotted onto solid TY medium containing either (50 g/L) sucrose, raffinose, glucose, or fructose, and incubated at 28 °C for 24 h.

Previously, *G. japonicus* LASM12 was shown to produce EPS in a sucrose-dependent manner similar to other isolates from sugarcane factories such as *Leuconostoc* sp. [3]. However, the further spotting assays of the *G. japonicus* strains LASM11 and LASM12 on solid TY medium containing either sucrose, raffinose, glucose, or fructose for the qualitative analysis of EPS production showed that the EPS is produced not only on sucrose, but also on raffinose, which indicates levansucrase activity (Figure 1). Sucrose is a disaccharide composed of glucose α-1,β-2-fructose while raffinose is a trisaccharide composed of galactose-α-1,6-glucose-α-1,β-2-fructose. Raffinose has been used previously as a levansucrase substrate that dextransucrase enzymes are unable to utilize [16,43]. Furthermore, as expected, no EPS was synthesized on either glucose or fructose monosaccharide as a carbon source.

### 3.3. Identification of Exopolysaccharide-Forming Enzymes

**Figure 2:**
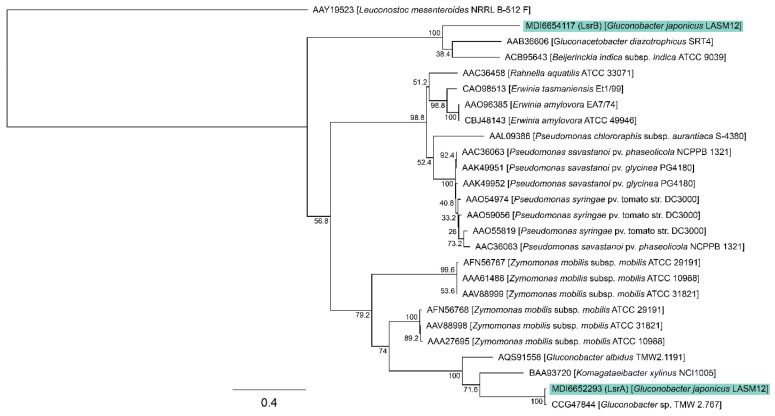
Maximum likelihood tree of putative levansucrase enzymes identified from *G. japonicus* LASM12 (highlighted in green) and characterized GH68 enzymes from Gram-negative bacteria in the CAZy database. The percentage of 500 bootstraps that supported each branch are indicated, and the scale bar represents the number of substitutions per site.

We next sought to detect and identify the enzyme responsible for EPS synthesis in the *G. japonicus* strain LASM12. The draft genome of strain LASM12 was searched by BLAST [44] using the sequence of the levansucrase enzyme encoded by the *Gluconobacter albidus* strain TMW2.1191 [6], which revealed two putative levansucrase genes with locus tags QMA67_04940 and QMA67_14420, predicted to encode 49 kDa and 65 kDa proteins, respectively. The products of these genes have been named LsrA and LsrB, respectively, and can be found in GenBank with the accession numbers MDI6652293 and MDI6654117. Interestingly, these proteins shared only 26.37% sequence identity. Despite the significant sequence diversity among the members of the GH68 glycoside hydrolase enzyme family (to which levansucrases belong), the sequence alignment and phylogenetic analysis showed that both the LASM12 putative levansucrases had high similarity to the characterized levansucrases, strongly supporting their predicted function (Figure 2). Further support for their predicted function came from examining the sequence alignment of both the putative LASM12 levansucrases against the characterized levansucrases, which showed that all the key substrate binding sites and catalytic residues are conserved (Supplemental Figure S1).

**Figure 3:**
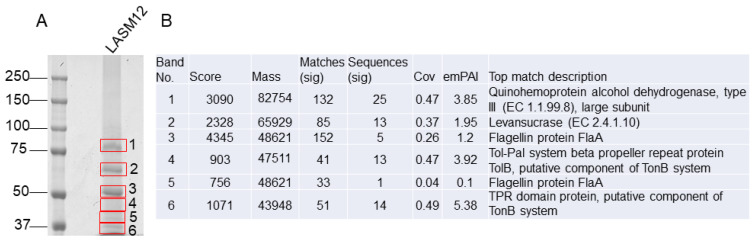
Identification of levansucrase enzyme in the secreted protein fraction of *G. japonicus* strain LASM12. Secreted protein fraction samples were resolved under reducing conditions on an 8% tris-glycine SDS-PAGE gel in panel (**A**), left. Bands were excised (red boxes), in-gel trypsin-digested, analyzed by QTOF LC-MS. The data shown is representative of three experiments. Peptides were analyzed twice; MS/MS precursor exclusion lists were generated for the second analyses to improve protein identification. Data were processed and searched using Mascot Distiller and a database constructed from the *G. japonicus* LASM12 genome. Top matches identified from each band are shown in panel (**B**), right.

To determine whether either of the levansucrase proteins are secreted into culture supernatants, 72 h culture supernatants were concentrated and resolved on a 1-D SDS-PAGE gel for band excision and identification. The LC-MS spectra of tryptic peptides were searched against a database constructed from the *G. japonicus* LASM12 genome. Searches revealed the presence of one levansucrase in band 2 as the protein with the highest score migrating at 65 kDa corresponding with LsrB (Figure 3). We observed 13 peptide matches to LsrB representing 37% of the predicted protein sequence (Figure 3). The identity of the top scoring proteins in bands 1–6 are listed in Figure 3. LsrB has a predicted mass of 65,929 Da prior to any cleavage of the predicted signal peptide of 27 amino acids (Pred-TAT) [45]. However, a signal peptide was not detected by Pred-TAT in the shorter LsrA protein (49,570 Da) sequence, which we were unable to detect in the secreted protein sample. Furthermore, a transmembrane domain was predicted for LsrB but not for LsrA (TMHMM) [46]. Therefore, we believe it may be unlikely that the latter levansucrase would be localized to the bacterial cell wall. Interestingly, another study of LevS from *Gluconobacter japonicus* LMG 1417 was reported to secrete a highly active levansucrase via a signal-independent pathway [47]. Likewise, we considered whether LsrA from *G. japonicus* LASM12 may also be secreted in the same manner without utilizing a signal peptide. However, despite multiple attempts to detect LsrA in the intracellular, cell wall, or secreted fractions, LsrA could not be detected. If present, LsrA may be below the limit of detection or produced under different experimental conditions. Taken together, it seems likely that LsrB is the primary levansucrase producing EPS in our experiments.

### 3.4. Structural Analysis of G. japonicus Exopolysaccharide

**Figure 4:**
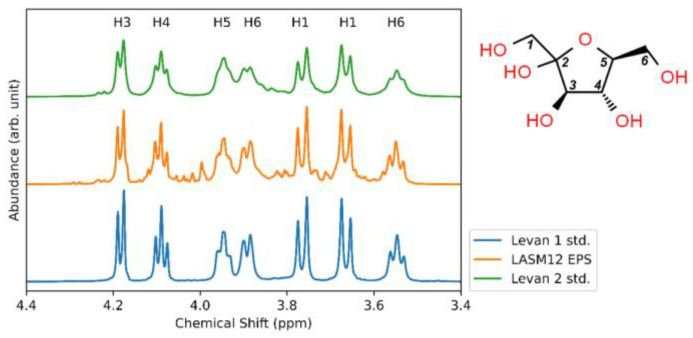
^1^H-NMR analysis of samples for (bottom to top) levan fructan standard from Timothy grass (Levan 1 std.), EPS from *G. japonicus* (LASM12 EPS), and levan fructan standard from *E. herbicola* (Levan 2 std.).

**Figure 5:**
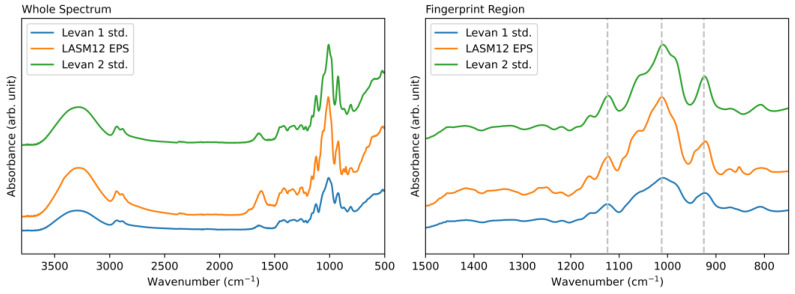
*G. japonicus* LASM12 EPS matches the peaks from commercial standards in the FTIR whole spectrum (**left**) and fingerprint region (**right**). Vertical lines in the fingerprint region subfigure correspond to fructan-specific peaks at 1124, 1012, and 925 cm^−1^.

The spotting assay of *G. japonicus* LASM12 showing sucrose- and raffinose-dependent EPS production (Figure 1) suggested levansucrase activity. Therefore, to further analyze the structure of *G. japonicus* LASM12 EPS, ^1^H-NMR was performed on the EPS sample and compared to two commercial levan fructan standards. The ^1^H-NMR and FTIR spectra are given in Figure 4 and Figure 5, respectively.

The NMR spectra between the commercial standards and analyzed LASM12 EPS were qualitatively equivalent. The peaks are all attributable to hydrogen signals in a repeating fructose monomer, which are labeled (1 to 6) in Figure 4 [48]. The minor peaks in the LASM12 EPS sample, which are absent in the standard levan samples, may be attributable to other sugars or sample contaminants, for which further analysis would be needed for fuller characterization. For the FTIR analysis, both the whole spectrum and fingerprint region for each sample are shown in Figure 5. There is qualitative correspondence between the LASM12 EPS and standard spectra, similar to the NMR results. All three FTIR spectra show a broad OH stretching peak near 3400 cm^−1^, and two smaller peaks near 2900 cm^−1^, which are attributed to asymmetric and symmetric methylene group vibrations [48]. Additionally, the peak near 1650 cm^−1^ is attributed to the characteristic carbonyl stretching vibration [49]. These four peaks have similarly been reported in previous work on levan characterization [49]. Three principal peaks (labeled with vertical lines) in the FTIR fingerprint plot are also attributable to features of a levan polysaccharide. The band at 1124 cm^−1^ is assigned to antisymmetric furan ring ether vibration, the band at 1012 cm^−1^ is assigned to alcoholic OH stretching, and the band at 925 cm^−1^ is assigned to the symmetric stretching of D-furanose [49]. These NMR and FTIR spectra provide robust evidence for confirmation that the LASM12 EPS sample is a levan fructan polysaccharide.

### 3.5. Glycosyl Monomer and Glycosyl Linkage Analysis of G. japonicus LASM12 Exopolysaccharide

**Figure 6:**
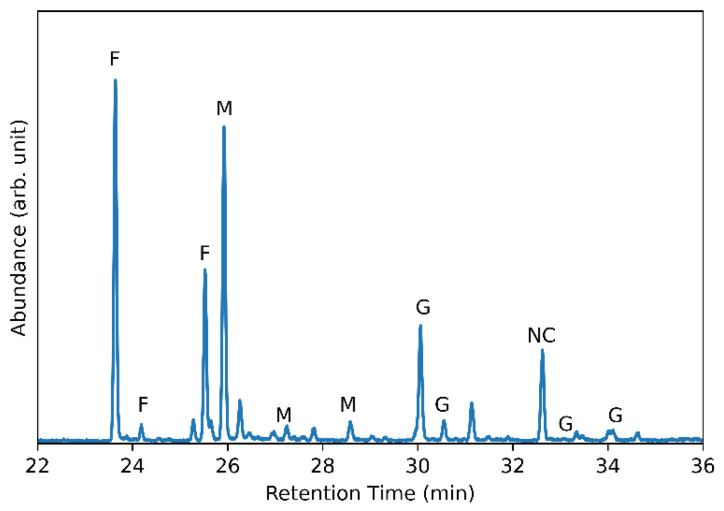
Glycosyl/monomer composition analysis gas chromatography-mass spectrometry (GC-MS) of *G. japonicus* EPS by per-O-trimethylsilyl (TMS) method. Peak labels denote either fructose (F), mannose (M), glucose (G) glycosyl residues, with one non-carbohydrate (NC) peak.

**Figure 7:**
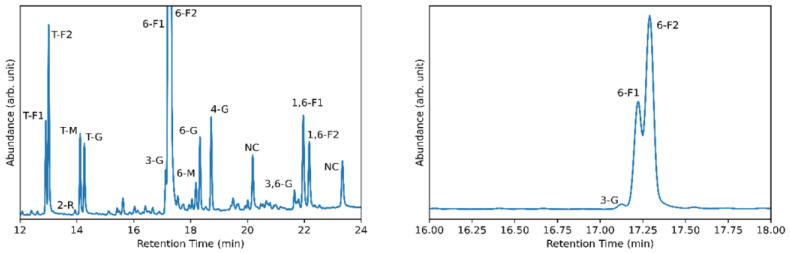
Glycosyl linkage analysis gas chromatography-mass spectrometry (GC-MS) of partially methylated alditol acetates (PMAAs) derivatives (**left**) and close-up to illustrate the larger relative abundance of 6-linked fructofuranosyl residues (**right**). Peak labels for terminal residues are denoted by a leading T; peak labels with leading numbers denote linkage locations; fructofuranosyl, rhamnopyranosyl, mannopyranosyl, glucopyranosyl, and non-carbohydrate residues are denoted by F, R, M, G, and NC labels, respectively; fructofuranosyl residues F1 and F2 correspond to isomers of glucitol and mannitol.

In addition to NMR and FTIR spectroscopic techniques, glycosyl monomer and glycosyl linkage analysis were also conducted. These results are shown in Figure 6 and Figure 7, respectively. The glycosyl residues were made up of 89.5 mol % fructose (F), 7.0 mol % mannose (M), and 3.5 mol % glucose (G). While the presence of mannose was unexpected, others have also reported the presence of mannose in levan-type fructans in which two molecular weight peaks were observed and the ratio of mannose:fructose:glucose was calculated and reported as 2.59:29.83:1 for EPS1 and 4.23:36.59:1 for EPS2 [50,51]. The ratio for the levan fructan produced by *G. japonicus* LASM12 was 2:25.6:1, which is very similar. The glycosyl linkage analysis shows that 75.1% of the analyzed sample is composed of 6-linked fructofuranosyl residues. The two peaks here (shown in Figure 7 (right)) are the result of the reduction of fructose during analysis, which produces two isomers (glucitol and mannitol). The terminal fructofuranosyl residues make up an additional 7.2% of the analyzed sample. These results are consistent with the majority of the analyzed sample being made up of 2,6-linked fructose units in a levan polysaccharide. The presence of 2,1-linked fructofuranosyl residues (5.7%) additionally suggests the presence of a small amount of branching in the sample.

### 3.6. Determination of Molecular Size of Exopolysaccharide Produced by G. japonicus LASM12

**Figure 8:**
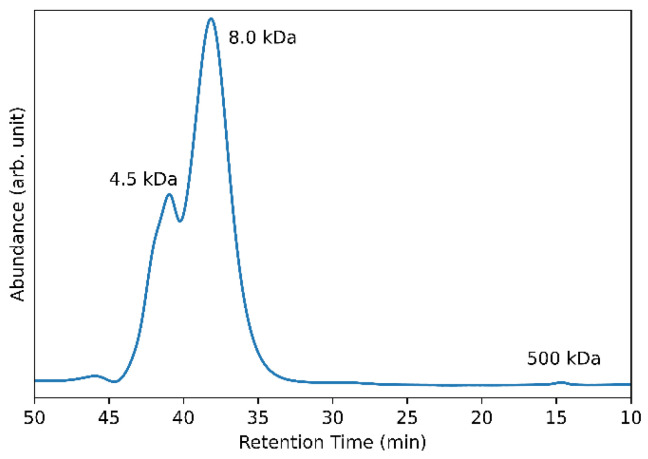
Gel permeation chromatography analysis indicates two bimodal primary peaks with retention times of 38.15 and 40.95 corresponding to 8000 and 4500 Da, respectively, as well as a minor peak of approximately 500 kDa at 15 min retention time.

Finally, gel permeation chromatography (GPC) was performed to determine the size of the EPS (Figure 8). The results from the GPC show that the analyzed EPS sample from *G. japonicus* LASM12 grown on sucrose had two main bimodal peaks with molecular weights of 4.5 kDa and 8.0 kDa. A very minor peak was also observed at 500 kDa.

## 4. Discussion and Conclusions

In this study, we identified and characterized the levansucrases and resulting levan fructan produced by *Gluconobacter japonicus* LASM12 that was previously isolated from a raw sugarcane processing facility in south Louisiana. *G. japonicus* LASM12 uses either sucrose or raffinose as a sugar substrate to synthesize EPS, which was demonstrated to be levan fructan polysaccharide by ^1^H-NMR and FTIR. Additional carbohydrate analytical chemistry indicated the EPS is composed of linear β-2,6-fructofuranosyl chains with occasional β-2,1-branching and the EPS polymers are present at 4,500 and 8,000 Da. These unusually small bimodal peaks may hold potential for biobased applications suited to shorter polymers. In contrast to this unusual size distribution of *G. japonicus* LASM12 EPS, those typically reported in the literature are a higher molecular weight. For instance, in their review on the production and characterization of microbial levans, Srikanth et al. summarized that levans are usually between 2 to 100 × 10^6^ Da [30]. Examples include *Bacillus mojavensis* (average size of 2.3 × 10^6^ Da) [52]; *Bacillus tequilensis*-GM (2.5 × 10^6^ Da) [53]; *Lactobacillus reuteri* FW2 (bimodal EPS with sizes at 12,000 Da and 2.4 × 10^6^ Da) [27]; and *Lactobacillus reuteri* LTH5448 (3.924 × 10^7^ Da) [28]. In sharp contrast, the lower-molecular-weight levan from *L. reuteri* FW2, at 12,000 Da, is more similar to the lower-molecular-weight EPS peaks we observed from *G. japonicus* LASM12 [27]. The fructan polymer yield, molecular weight, and degree of branching has been reported to be dependent on the levansucrase enzyme [29]. Other factors that may affect levan polymer length include temperature, enzyme concentration, and available substrate concentration during synthesis [54,55].

Further, in this study, we also describe the bioinformatic and proteomic detection of a levansucrase enzyme, LsrB. While the gene for LsrA is encoded in the genome, LsrA could not be detected in the intracellular, cell wall, or secreted fractions. If present, LsrA appears to be below the limit of detection or may be differentially produced under different growth conditions. Therefore, under the experimental conditions in our study, LsrB appears to be the most likely levansucrase involved in this levan fructan synthesis.

Levan polysaccharides have several potential applications (e.g., biotechnology, food and agriculture, cosmetics, pharmaceuticals), all providing a strong rationale for the broad interest in identifying and characterizing levansucrases and the resulting EPS products, which may be leveraged as microbial-produced bio-based products. Moreover, the microbiota of sugar crop processing factories may represent an abundant source of microorganisms producing novel or previously uncharacterized versions of carbohydrate-active enzymes such as levansucrases and dextransucrases that may be instrumental in future bio-based product design.

Further study of the *G. japonicus* LASM12 levan fructan physico-chemical properties such as the water-holding capacity and influence on viscosity may further illuminate whether fructans contribute to viscosity problems during sugar crop processing. It is also possible that fructan polymer molecular size affects the impact on viscosity, so larger-molecular-weight fructans should also be investigated. Additionally, future work is needed to examine whether levan fructan EPS may be a contributor to the problem of elongated sugar crystals during sucrose crystallization. This characterized levan fructan may be a useful reagent that can easily be produced by *Gluconobacter japonicus* LASM12 and used for future studies aimed at measuring the impact of fructans on sugar crop processing, extraction, and crystallization.

Finally, this work definitively illustrates the presence of levan-producing microorganisms during sugarcane processing for raw sugar manufacturing. Future work will explore the development of levans as bio-based products and mitigation strategies for levan fructans in sugar crop processing industries.

## Data Availability

Genomic data are available through the open access publication [9]. Experimental data can be made available upon request.

## Supplementary Materials

The following supporting information can be downloaded at https://www.mdpi.com/article/10.3390/microorganisms12010107/s1, Figure S1: Amino acid sequence alignment of putative levansucrase enzymes identified from LASM12 (names highlighted) and representative GH68 enzymes from Gram-negative bacteria in the CAZy database. Representative sequences were chosen such that all sequences are <97% identical to each other. The amino acid sequences were aligned with Clustal-Omega and the N- and C- terminal portions of the alignment were omitted due to low conservation. The consensus sequence above the alignment represents >50% conserved residues and the blue and underlined residues in the consensus sequence represent substrate binding sites and catalytic residues, respectively [56]. The conservation graph above the alignment shows the percent identity at each position.
