# Supplementary material for: Characterization of Levan Fructan Produced by a Gluconobacter japonicus Strain Isolated from a Sugarcane Processing Facility
Source: Microorganisms. 2024 Jan 5;12(1):107. doi: 10.3390/microorganisms12010107 (PMC10819292; doi:10.3390/microorganisms12010107)
Supplement: Supplementary file 1 [file microorganisms-12-00107-s001.zip › supplementary figure S1.pdf]

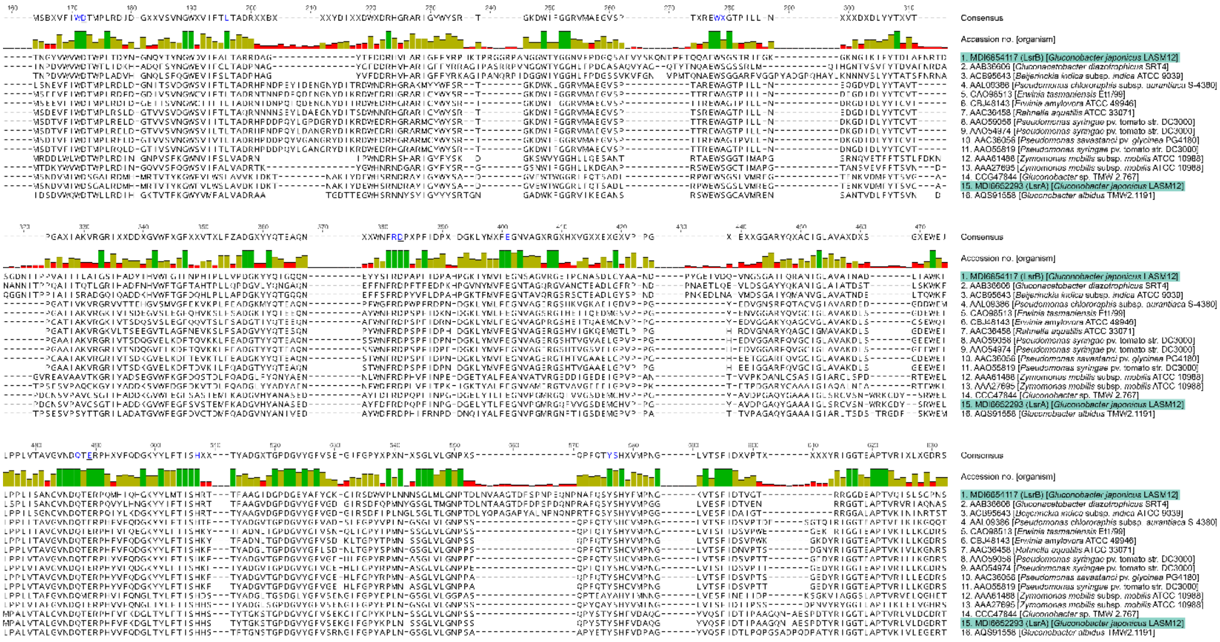

**Supplemental Figures.**

Supplemental Figure S1. Amino acid sequence alignment of putative levansucrase enzymes identified from LASM12 (names highlighted) and representative GH68 enzymes from Gram-negative bacteria in the CAZy database. Representative sequences were chosen such that all sequences are <97% identical to each other. The amino acid sequences were aligned with Clustal Omega and the N- and C- terminal portions of the alignment were omitted due to low conservation. The consensus sequence above the alignment represent >50% conserved residues and the blue and underlined residues in the consensus sequence represent substrate binding sites and catalytic residues, respectively [56]. The conservation graph above the alignment shows the percent identity at each position.
